# Post-trial access to and use of pre-exposure prophylaxis in Durban, South Africa

**DOI:** 10.1186/s12889-023-16139-z

**Published:** 2023-06-22

**Authors:** Ivana Beesham, Cecilia Milford, Jenni Smit, Dvora L. Joseph Davey, Jared M. Baeten, Renee Heffron, Mags Beksinska, Leila E. Mansoor

**Affiliations:** 1 Wits MatCH Research Unit (WMRU), Department of Obstetrics and Gynaecology, Faculty of Health Sciences, University of the Witwatersrand, Durban, South Africa; 2 Division of Epidemiology and Biostatistics, School of Public Health and Family Medicine, University of Cape Town, Cape Town, South Africa; 3 Division of Infectious Diseases, Geffen School of Medicine, University of California, Los Angeles, CA USA; 4 Department of Global Health, Department of Epidemiology, Department of Medicine, University of Washington, Seattle, WA USA; 5 Gilead Sciences, Foster City, CA USA; 6 University of Alabama at Birmingham, Alabama, USA; 7 Department of Global Health and Department of Epidemiology, University of Washington, Seattle, WA USA; 8 Centre for the AIDS Programme of Research in South Africa (CAPRISA), University of KwaZulu-Natal, Durban, South Africa

**Keywords:** Oral pre-exposure prophylaxis, Post-trial access and use, Young women, South Africa

## Abstract

**Background:**

HIV endpoint-driven clinical trials increasingly provide oral pre-exposure prophylaxis (PrEP) as standard of prevention during the trial, however, among participants desiring to continue using PrEP at trial exit, little is known about post-trial PrEP access and continued use.

**Methods:**

We conducted one-time, semi-structured, face-to-face, in-depth interviews with 13 women from Durban, South Africa, from November to December 2021. We interviewed women who initiated oral PrEP as part of the HIV prevention package during the Evidence for Contraceptive Options and HIV Outcomes (ECHO) Trial, elected to continue using PrEP at study exit, and were given a 3-month PrEP supply and referred to facilities for PrEP refills at the final trial visit. The interview guide probed for barriers and enablers to post-trial PrEP access, and current and future PrEP use. Interviews were audio-recorded and transcribed. Thematic analysis was facilitated using NVivo.

**Results:**

Of the 13 women, six accessed oral PrEP post-trial exit, but five later discontinued. The remaining seven women did not access PrEP. Barriers to post-trial PrEP access and continued use included PrEP facilities having long queues, inconvenient operating hours, and being located far from women’s homes. Some women were unable to afford transport costs to collect PrEP. Two women reported visiting their local clinics and requesting PrEP but were informed that PrEP was unavailable at the clinic. Only one woman was still using PrEP at the time of the interview. She reported that the PrEP facility was located close to her home, staff were friendly, and PrEP education and counselling were provided. Most women not on PrEP reported wanting to use it again, particularly if barriers to access could be alleviated and PrEP was easily available at facilities.

**Conclusions:**

We identified several barriers to post-trial PrEP access. Strategies to enhance PrEP access such as a reduction in waiting queues, convenient facility operating hours, and making PrEP more widely available and accessible are needed. It is also worth noting that oral PrEP access has expanded in South Africa from 2018 till now and this could improve access to PrEP for participants exiting trials who desire to continue PrEP.

## Background

Recently, several HIV prevention and endpoint-driven clinical trials have provided tenofovir-based oral pre-exposure prophylaxis (PrEP) to study participants as a component of their standard package of HIV prevention [1–3]. This has been driven partly by ethical guidelines that recommend that participants enrolling in HIV prevention research should be provided with a package of HIV prevention methods recommended by the World Health Organization (WHO), but also that prevention services should be effective, comprehensive and sustainable [4]. This is of importance as prevention research trials frequently enrol populations who are at elevated risk of HIV acquisition, including adolescent girls and young women (AGYW) [5].

An additional consideration for the provision of oral PrEP in clinical trials is assessing what prevention services are available to the local community and how this differs from the prevention package provided during the trial [4]. When the South African Medical Research Council (SAMRC) first recommended the provision of oral PrEP in HIV prevention trials in 2017, it recognized that at the time, oral PrEP availability was limited in South Africa with only 7,000 people accessing oral PrEP, and the importance of increasing oral PrEP access [6]. Access to effective HIV prevention and treatment modalities post-trial termination is important to ensure sustainability and continued use for participants who desire to continue using study drugs after exiting the trial.

In South Africa, the rollout of oral PrEP began in 2016 at select facilities that provided services to sex workers [7], expanding in 2017 to include men who have sex with men (MSM) and university students [8]. In early 2018, oral PrEP was gradually rolled out to AGYW at primary health care facilities [8]. PrEP guidelines in South Africa were updated in 2020 to include additional populations considered to be at “substantial risk” for HIV infection which included, in addition to the populations described above, people with more than one sexual partner, people who inject drugs, those with recent sexually transmitted infections (STIs), serodiscordant couples, and those who recognized their own risk and requested oral PrEP [9]. Finally, in 2021, further updates were made to the South African PrEP guidelines which included offering oral PrEP to pregnant and lactating women [10]. As of July 2022, cumulative oral PrEP initiations in South Africa were estimated to be ~ 530,000 [11]. Oral PrEP availability is an essential component of PrEP access, however, several other factors may impede PrEP access, such as distance and travel costs to attend clinics, having to take time off work/school for clinic appointments, long queues and inconvenient operating hours at public health facilities, stigma, eligibility criteria, and limited service delivery models [12–14].

While several studies have described oral PrEP uptake and persistence [12, 15], data on oral PrEP access and use post-trial is sparse. In this qualitative study, we describe post-trial oral PrEP access and use among women from Durban, South Africa who initiated oral PrEP at a research clinic as part of the HIV prevention package provided through the Evidence for Contraceptive Options and HIV Outcomes (ECHO) Trial [2] and elected to continue using oral PrEP at trial exit. Participants were referred to facilities for oral PrEP refills. We also describe HIV testing and family planning use post-trial, and suggestions by women to improve oral PrEP services in their communities.

## Methods

### ECHO Trial overview and procedures

The ECHO Trial was a randomized clinical trial, conducted from 2015 to 2018, in four African countries (Eswatini, Kenya, South Africa and Zambia). HIV-uninfected women, aged 16–35 years and desiring contraception, were enrolled and randomized to one of three effective contraceptives [2]. The primary study endpoint was HIV incidence. Follow-up was quarterly for 12–18 months. Detailed ECHO Trial procedures and the primary trial results have been published elsewhere [2].

### Oral PrEP provision during the ECHO Trial

During the latter eight months of the trial (March to October 2018), oral PrEP was provided onsite by trial staff at the South African trial sites [16]. Staff that were involved in PrEP provision included pharmacists, counsellors, and outreach, administrative, clinical (doctors and nurses) and quality assurance staff (data teams) [16]. Women who initiated oral PrEP onsite at trial sites and requested to continue using oral PrEP at trial termination were given a three-month supply of oral PrEP at the final study visit and referred to off-site facilities for ongoing oral PrEP access.

### Current study procedures

For this study, we recruited women who had participated at the trial site in Durban, South Africa, who had had initiated oral PrEP onsite during the ECHO Trial and continued using oral PrEP at trial exit. All these women had provided written informed consent to be contacted for future studies. An ancillary study was conducted at this research site to quantitatively explore PrEP use during the ECHO Trial and assess PrEP use, PrEP discontinuation, and reasons for discontinuing six months after exiting the trial [17]. We purposively recruited women who participated in the ancillary study who reported continuing oral PrEP at trial exit and had 1) accessed oral PrEP post-trial exit and either continued or discontinued at the time of the interview and, 2) did not access oral PrEP after exiting the trial. Eligible women were contacted by telephone and invited to participate in a one-time, in-person, in-depth interview. A maximum of two follow-up telephonic calls were made to women who did not respond on the first attempt. Interviews were conducted from November to December 2021. The semi-structured interview guide explored reasons for initiating oral PrEP during the trial, oral PrEP use following study exit, barriers and enablers to oral PrEP access post-trial, reasons for oral PrEP discontinuation if applicable, and suggestions to improve oral PrEP services for women.

Interviews were conducted in a quiet, private room at the research site to ensure confidentiality. They were conducted by a female interviewer (IB) in English. Interviews were conducted in English as all women who participated in the ancillary study chose English as a language of choice when offered a choice between English and isiZulu, and in general women were fluent in both languages. IB is a medical doctor and has 7-years of experience in conducting research. Women were informed that IB was conducting this research as part of her PhD program. IB was a study clinician in the ECHO Trial, was known to the participants, and had provided clinical care to several participants during the ECHO Trial, including the provision of oral PrEP. IB is also of a similar age group to the participants. All interviews were conducted approximately three years after the ECHO Trial had ended. Interviews lasted approximately 15 to 30 min.

All interviews were audio recorded and transcribed verbatim. Field notes were taken during the interview. A thematic approach was used for coding and analysis of interviews [18]. Analysis was facilitated using NVivo (QSR International, version 10). An initial code list was developed which was modified and refined as additional transcripts were coded. The code list and coding were reviewed by a qualitative researcher (CM). The codebook was developed inductively and deductively, and was informed by thematic areas outlined in the interview guide as well as participant responses. We selected representative quotes from women to highlight some of the study findings, and present these with the participant’s current age (at the time of the interview).

As interviews were conducted during the COVID-19 lockdown period in South Africa, measures were put in place at the research site to reduce the risk of COVID-19 transmission. These included screening all staff and participants for signs and symptoms of COVID-19, ensuring staff and participants wore face masks, practicing physical distancing, ventilating consulting rooms, frequent hand sanitization, and having physical screens between staff and participants. The lockdown alert level at the time (alert level 1) permitted most activities to resume including movement of people.

### Ethics

This study was approved by the University of the Witwatersrand’s Human Research Ethics Committee (Wits HREC) (Reference: M191155). All participants signed written informed consent in English to participate in the qualitative post-trial access in-depth interviews. Participants were reimbursed ZAR200 (approximately USD12) for their time and transport costs. The ECHO Trial was approved by Wits HREC (Reference: 141112) and FHI360. All methods were carried out in accordance with the Declaration of Helsinki.

## Results

We attempted to contact 26 eligible women who had elected to continue oral PrEP at ECHO Trial exit. Of these, 13 were contactable via telephone and agreed to be interviewed (50% acceptance rate), one (4%) had relocated to another province and reported she was unable to attend an in-person interview (declined participation), and 12 (46%) were not contactable via telephone. Demographics at enrolment into the ECHO Trial are presented in Table 1. At the time of the interview, the median age of women was 26 years (range: 22 to 30 years).

**Table 1:** Participant demographics at enrolment into the ECHO Trial^a^

Characteristic	n (%) or median (range)
Median Age (range) (years)	22 (18 to 25)
Marital Status	
Never married	13 (100)
Education	
Secondary school (incomplete)	5 (38)
Secondary school (complete)	5 (38)
Post-secondary school	3 (23)
Lives with Partner	
Yes	1 (8)
No	12 (92)
Earns own income	
Yes	4 (31)
No	9 (69)

^a^ Characteristics were collected at enrolment in the ECHO Trial and not repeated at the time of the interview. Demographics refer to the 13 women enrolled in this post-trial access study

### Reasons for initiating oral PrEP during the ECHO Trial

Women reported several reasons for electing to use oral PrEP during the ECHO Trial (Table 2). These included inconsistent or non-use of condoms, partner-related reasons such as not trusting the partner, wanting to protect oneself from acquiring HIV, and engaging in risky sexual behavior. Drivers of condom inconsistency/non-use included the partner not wanting to use condoms, using condoms with some partners but not others, and not enjoying sex when using condoms.

“The reason that [I] decide that I’m using the PrEP was just make sure my to be safe for myself … because I don’t trust my boyfriend most of the time.” (26 years)

**Table 2:** Reasons reported by women for initiating oral pre-exposure prophylaxis (PrEP) during the ECHO Trial

Reasons for using oral PrEP	Quotes
Condom-related	
Inconsistent or non-use of condoms Fear that condoms might “break” during sex	“I decide to use PrEP because most of the time when I’m doing sex with my partner we don’t use condom.” (23 years) “Uhm I decided to use PrEP because uh sometimes uhm as we are teenagers let me uh just not say we teenagers, like we [are] girls … We don’t like condoms” (22 years) “My decision to use PrEP … is in order to protect myself because we know that you can use your condom but it’s not 100% sure it can break.” (24 years)
Partner related	
Does not trust partner Partner is unfaithful	“reason that decide that I’m using the PrEP was just make sure my, to be safe for myself … Because I don’t trust my boyfriend most of the time.” (26 years) “the reason why I decided to use PrEP it’s because they said it will prevent HIV even if your partner is not faithful” (29 years)
To prevent/protect oneself from acquiring HIV and/or to feel safe	“because I saw the whole benefit of it [PrEP] … it help you to prevent HIV and AIDS” (26 years) “I want to be to be protected that’s why I decide to use PrEP” (23 years)
Gender stereotypes	“You cannot cannot trust anyone in this world especially mens you cannot trust them … those people go around sleep with everyone” (27 years) “boys they playing so much … And so I decide to take PrEP” (26 years)
Engaging in risky behavior	“I like enjoying party, so you know what happened (laughs) we got drunk … Sometimes they new people, you know one night stand … I sleep with maybe two … two different guy[s] in the one day” (28 years)
Planning for pregnancy	“I think for the future reason … I also need to prevent [HIV] … because … I don’t want to to infect my child once I’m get pregnant” (30 years)
Other	“Okay coz I came here [to research site] and then you’ll introduced me to PrEP and you’ll told me like the good and the bad about it, and I decided to take it coz it prevents you from being affected and it helps you … You may never know you might get raped someday you can never plan for HIV (laughs) … I need to make sure that like I don’t have, if I were to get affected it must be like not on purpose like accidently like when we getting raped or getting like, I don’t know how else you get (laughs) HIV (laughs)” (27 years)

### Disclosure of oral PrEP use

All women in our study reported disclosing oral PrEP use. Women reported disclosing to family members, friends, partners and community members. A few women reported not disclosing to their partners, even though they had disclosed to others such as family members. Reasons for withholding disclosure from the partner included losing the partner’s trust in her and/or an assumption she is unfaithful; the partner’s confusion that the PrEP pills are ART; and anticipating a negative reaction from the partner, for example one woman reported her partner was unhappy about her using an intrauterine contraceptive device, and upon disclosure to her partner, he insisted that she have it removed, so she felt her partner might similarly react negatively to her using PrEP. Many women reported that those to whom they had disclosed had positive reactions and/or supported their PrEP use. Few women reported experiencing negative reactions to disclosing PrEP use, however they felt that this did not impact on their PrEP use.

“I told them [cohabiting family members]. People were like, they were proud of me [for taking PrEP], they thought that I was like, that was a good thing to do and that that is a mature thing to do.” (27 years)

### Oral PrEP continuation and use post ECHO Trial exit

**Fig. 1:**
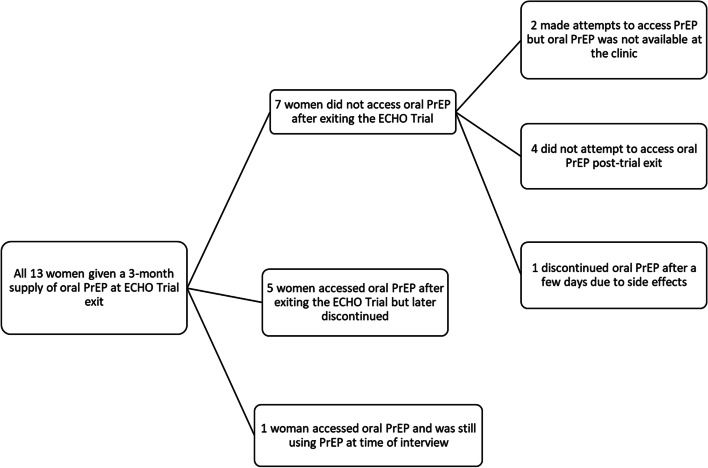
Post-trial oral pre-exposure prophylaxis (PrEP) access and use among women from Durban, South Africa

Some women reported accessing oral PrEP post-trial exit, while other women did not (Fig. 1). The duration of oral PrEP use after exiting the study varied. Among women who reported not accessing PrEP post-trial termination, almost all reported completing the three-month oral PrEP supply that was provided at ECHO trial exit. One woman reported starting oral PrEP after the study had ended, and then discontinued PrEP after 3–4 days due to side effects. This woman reported that she had been given oral PrEP during the ECHO Trial, and continued oral PrEP at trial exit, however, she reported in the post-trial interview that she only took her first PrEP dose after the study had ended. Among women who reported accessing PrEP after exiting the study and then discontinuing, the frequency of oral PrEP refill collection ranged from a single collection to five collections, and the maximum duration of oral PrEP use post study exit was approximately one-year. Finally, one woman who was still using oral PrEP at the time of the interview reported continuous use of oral PrEP for more than 3 years post study.

### Oral PrEP adherence post ECHO Trial

Most women reported missing doses while using PrEP. Reasons for missing oral PrEP doses included forgetfulness, being away from home when oral PrEP needed to be taken and not having PrEP pills with them, and fear of what others would think of her taking PrEP.

“[I]t is difficult I won’t lie because sometimes you forget and sometimes … you go to work and forget them at home.” (24 years)

“Maybe if I’m going out with my friends … I don’t take it [oral PrEP] sometimes … maybe I’m scared if they were saying how I’m eating [why am I taking pills] … But when I’m at home I’m actually make sure I’m drinking [taking PrEP].” (26 years)

Women reported several different methods that facilitated adherence, and in some cases, women used more than one method. These included setting an alarm as a reminder to take oral PrEP, taking oral PrEP when a cohabiting family member takes chronic treatment, storing PrEP pills in a visible area, support from family members to take oral PrEP, and remembering to take PrEP pills when away from home.

“I’m not forgetting to [take] the pills … my [family member] is HIV positive … so when they, when my [family member] take that pills and I will take mine [PrEP pills].” (28 years)

One woman reported sharing PrEP pills with her partner and storing a bottle of oral PrEP at his place.

“[H]e [partner] was sharing it [PrEP] … I give him one bottle [of PrEP tablets] … When I’m going to his house, I’m using it [PrEP tablets] and he’s also using it [her PrEP pills].” (27 years)

### Barriers to post-trial oral PrEP access and continuation

Women identified several barriers to accessing oral PrEP after exiting the ECHO Trial, resulting in discontinuation of oral PrEP use. These barriers included having to spend too much time at the clinic due to long queues, clinics being located far away from women’s homes, and/or being unable to afford transport costs to access clinics.

“The problem is that the clinic near my home … they have long lines so … I can’t wait … there’s a long queue to get in the clinic … that’s why I stopped taking it [oral PrEP].” (23 years)

“The place to take [collect] the PrEP which was far from home … that’s why I didn’t take it … it was difficult because I had no money … it will take 2 taxi … I think it was uh ZAR100 [approximately USD6] return.” (26 years)

Two women reported that they went to their local clinic and requested oral PrEP, however it was not available.

“I stopped using PrEP after the study ended because it’s very difficult to get PrEP. Most of the clinics they don’t have it.” (27 years).

Other barriers to oral PrEP access and continued use included being unable to attend clinic visits due to work commitments, and provider-related factors such as a lack of confidentiality.

“They [PrEP provider at clinic] don’t do their job properly … like when I’m talking to you [research staff] it’s confidential … They [PrEP provider at clinic] don’t keep that that thing, they shout, “[name of person], come here” and tell you this and this and this about the patient. I’m here, that … don’t make me comfortable.” (27 years)

### Facilitators and barriers to oral PrEP access after exiting the ECHO Trial

Women who accessed oral PrEP post-trial exit reported factors which facilitated their oral PrEP access and use. These facilitators included that the clinics providing oral PrEP were located close to their home, not having to wait in queues for their PrEP, and accessing free transport from the research site to the clinic.

“It’s [the clinic] just a walk it’s not far you don’t have to take a taxi there.” (24 years)

“It [collecting PrEP] was quite quick, quick and easy. There wasn’t any queue.” (30 years)

Many women also reported that the referral letter provided by the research site facilitated ease of access to oral PrEP services at the clinic.

“Yes I did [take the referral letter to the clinic] … other than that they [clinic staff] wouldn’t accept me or what they will have to ask me some more questions.” (24 years)

Other factors reported by a few women that facilitated oral PrEP access and use included quality information and counselling provided by clinic staff, oral PrEP demand creation, and friendly and welcoming clinic staff.

“I think PrEP is now like exposed in many clinics … our clinic … they give a lot of information about it [PrEP]. They teach about it [PrEP], like everything. Every morning if someone goes to our clinic it will be the same thing every morning they update … they always give updates about PrEP.” (24 years)

Despite some women accessing oral PrEP after exiting the study, many of these women reported barriers to continued use. These barriers were similar to those experienced by those women who had not accessed oral PrEP after exiting the main study. They included oral PrEP facilities being located far from their homes, public transport not utilizing routes where oral PrEP facilities were located, long waiting queues, unsuitable clinic operating hours due to work commitments, and provider-related factors.

“At the clinics they don’t attend us well … they don’t treat us they don’t trust us treat us like you do here [at the research site]. Sometimes we are not comfortable sometimes the nurse will shout at you so you end up not having appetite to go and fetch the uh PrEP at clinics.” (26 years)

“It’s hard because eh the queue is long you have to stay three hours to four hours waiting for you to call you ... you had to wait three hours because you are in the same queue with the people who are sick, with the people who are going to do this and that.” (24 years)

“I work every day. I only get one [day] off and the off I get it’s on weekends the clinics are closed.” (24 years)

One woman reported that not having a referral letter was a barrier to oral PrEP access – she reported that she had decided to transfer from one PrEP facility to another located closer to her home, but when she arrived at the more convenient PrEP facility, clinic staff requested a referral letter.

“But was struggling to get it [oral PrEP] because it [PrEP facility] was far … there by [name and location of PrEP facility] … Yes it was far for me to go there when I try to go get it here when it was down here [different PrEP facility] … they said I must go and get a referral letter. So I wasn’t happy to get one [referral letter] again.” (30 years)

Finally, one woman reported that although she had accessed oral PrEP easily at a local clinic that was located close to her home, and that it was a pleasant experience, she had decided to discontinue oral PrEP use without any reason.

“Ja it was my decision I just I decided to just stop I don’t know why.” (29 years)

### Integration of oral PrEP with other services

Most of the women who accessed oral PrEP after exiting the study had utilized public sector clinics. Women reported that services such as HIV testing, family planning and primary health care were available at the clinics, but frequently, these were not provided by the same provider or were in different locations within the same facility.

Interviewer: “And tell me do you get all this [family planning, HIV testing, primary health care services] at the same place where you get your PrEP, so your family planning and your HIV test, is that done there or is it done at a different place in the clinic?”

24-year female: “Everything has its door. There is a door for testing, there is a door for family planning, and there is a door for pills collecting.”

Interviewer: “Okay … So it’s different people that do each of these things?”

24-year female: “Yes.”

### Family planning access post-trial exit

Some women reported that they continued using their intrauterine devices and contraceptive implants that were inserted during the ECHO Trial, and therefore, did not need to access contraception after exiting the trial. Other women reported accessing their local clinics for injectable contraceptives, however, oral PrEP was frequently not available at facilities women used for family planning.

### HIV testing post study exit

Most women reported accessing HIV testing after exiting the study. HIV testing was most frequently accessed at clinics, but some women also reported using mobile HIV testing services, hospitals and blood donor services to test for HIV. Many women reported that they tested negative for HIV after exiting the trial. They reported that oral PrEP was not usually discussed at HIV testing, and often, was unavailable at the same facility where HIV testing was done. None of the women reported testing positive for HIV after exiting the trial.

Interviewer: “Okay did you do HIV testing afterwards [after the study ended]?”

26-year female: “Yes, I did.”

Interviewer: “Where did you test?”

26-year female: “At the clinic at the other side but they didn’t tell me about PrEP.”

### Future PrEP use

Among women not using oral PrEP at the time of the interview, almost all reported they would like to use oral PrEP in future, particularly if barriers to access could be removed.

“Yes I couldn’t get transport … So it was not easy to me to come back and go there … the transport is far so there I was stopping to eat PrEP … but if you can say I can go to fetch PrEP I can even go if there is a transport I can go to fetch PrEP.” (26 years)

### Suggestions to improve PrEP services

Women provided several suggestions on ways to improve oral PrEP services and make these more accessible (Table 3). These included increasing knowledge and awareness on oral PrEP among oral PrEP users, their family members, communities, and healthcare providers. Women also reported that oral PrEP needs to be made readily available at clinics. Some women suggested more convenient clinic operating hours for example, clinics to open for oral PrEP services over weekends, and a reduction in queues for oral PrEP refills. The integration of oral PrEP delivery with other services such as pharmacies, the provision of longer-acting PrEP methods, and the provision of oral PrEP in a trial/research setting were also suggested as potential ways of improving oral PrEP services and use for women.

**Table 3:** Suggestions provided by women on improving pre-exposure prophylaxis (PrEP) services

Suggestion(s)	Quotes
Increasing awareness and knowledge about oral PrEP among users, and increased availability of oral PrEP	“People will get education more and more and more about it [PrEP] like I think from clinics they need to be sent the PrEP, needs to go the clinics and then those, the nurses need to tell people about it … in the clinics they need to educate people about it … Even old people, tell them about it, they’ll go home and they’ll tell their children and the news will go on like that like that” (27 years) “That they should like … maybe they should tell the nurses at the clinic like that PrEP should be everywhere, educate people at the clinics. You see like condoms, every clinic has a condom. So every clinic should have uh PrEP.” (27 years)
Increasing knowledge and awareness about oral PrEP among providers	“Like there’s this like clinic, they [clinic staff] didn’t even know about it. I was like no, I had to google it, I was like “this here” [showing clinic staff]. They [clinic staff] like, “No, we, we don’t have that here.” (27 years)
Reduction in waiting queues and/or separate queues at the clinic for oral PrEP	“I think at the clinics they should provide the place if you come at the gate … some certain like uh uh it’s uh sort of a timetable … if you are going for PrEP you go that side, if you are going for this you go this side, so it will be easy for you if you go to the clinic if you know *uthi* (isiZulu word -says) you are supposed to [go to] that door rather than standing in one queue with the people who are go have different places with you” (24 years)
Availability of longer-acting PrEP modalities	“Maybe if they can be injections, not pills … maybe people can more women can try … can do PrEP because it’s hard to take a pill everyday (sighs) … It’s hard it’s kind of hard” (29 years)
Integrating oral PrEP delivery	“Pharmacies, they can also give PrEP because it will be easy if you have a a pharmacy next to you, you can go there and look for PrEP … even if you are at work, you you don’t have PrEP at that time, you can go to the clinic [at the pharmacy] and ask for PrEP” (24 years)
Reminders for clinic appointments	“Maybe if there could be another place that’s based on that where they gonna call and remind you, “Do you still remember you have to come and take PrEP?” Yes because sometimes we have a lot of things we that we are doing … we go to school we are working we have to go home and all those thing maybe you even forget … you even forget your date” (27 years)
Provision of oral PrEP in a research / trial setting	Interviewer: What do you think would make it easier for women to get PrEP when studies finish? 27-year female: It’s to continue with the study … It’s to continue with the study. That’s what I can say … Very easy and comfortable [to get oral PrEP as part of the study]. We always feel like free. Here it’s, it’s more like you were going to your own personal doctor “I don’t know how it can happen if we can have like, like uh study that basically for PrEP like how you [research site staff] uh get hold of us try to get hold of more women get them here make them sit down like how you sat down with us [ECHO Trial participants] and tell them about the PREP maybe it’s gonna make a difference. Maybe after like uh uh three months or six months you can refer them to a clinic. Then a person is used to the se uh side effects and they know what they are doing then they can for sure you can go to the clinic this will take it when someone sat you down and told you about it. (24 years)
Clinics to provide PrEP after-hours or on weekends	“It will be easy, especially weekend time [for clinics to provide oral PrEP] … Some people they work, they are working during week” (27 years)

## Discussion

In our study which explored post-trial oral PrEP access and use among women from Durban, South Africa who elected to continue using oral PrEP at ECHO study exit, we found that women experienced several barriers which limited their continued use of PrEP. Barriers included long queues at PrEP facilities, facilities being located far from women’s homes, being unable to afford transport costs to access clinics, unsuitable clinic operating hours, negative attitudes from providers, and oral PrEP being unavailable at some facilities. Conversely, women reported facilitators to oral PrEP access and use, such as clinics being located close to their homes, quality information and counselling provided by PrEP providers, oral PrEP demand creation and having a referral letter from the research site to collect oral PrEP refills. Despite only one woman reporting ongoing oral PrEP use at the time of the interview, most women expressed a desire to use oral PrEP again, particularly if barriers to access could be removed. To our knowledge, our study is one of the first studies to report on post-trial oral PrEP access among women who used oral PrEP as standard of care for HIV prevention in a trial setting.

Our study findings should be interpreted in conjunction with the rollout of oral PrEP in South Africa. In 2018, when study participants on oral PrEP began exiting the ECHO Trial, oral PrEP in the public sector in South Africa was being rolled out to sex workers, MSM, university students and AGYW, and in that year, total PrEP initiations were only 8,593 and PrEP was available at only 73 sites nationally [19]. This could account for the limited availability of oral PrEP reported by women in our study, as well as the long distances women had to travel to access oral PrEP. The scale-up of oral PrEP in South Africa in primary health clinics began late in 2019, and as of April 2022, PrEP was available at 2,915 facilities and more than 500,000 individuals had initiated oral PrEP [19]. However, despite the increased availability of oral PrEP, stock-outs of oral PrEP have been reported [20]. Further challenges with the rollout of oral PrEP in South Africa include that oral PrEP was first rolled in stigmatized populations, a lack of marketing and demand creation during the early stages of rollout, frequent follow-up visits and laboratory monitoring and the need for specialized staff to prescribe PrEP – all of which posed barriers to increased uptake and scale-up of PrEP.

Barriers to oral PrEP access reported in our study have been consistent with findings from studies conducted in Africa [12, 21, 22]. A study conducted among AGYW in Kenya and South Africa found that barriers to oral PrEP persistence included being unable to collect PrEP refills due to school/work commitments, and lacking transport to fetch PrEP from distant clinics [12]. Similarly, a study conducted in Zimbabwe among clients attending a family planning clinic and a youth facility, found that unsuitable clinic operating hours and being unable to afford transport costs to PrEP facilities were barriers to continued PrEP use [22]. In a study conducted among males and females aged 13–24 years in South Africa, Uganda and Zimbabwe, long waiting queues and negative attitudes of healthcare workers were perceived barriers to oral PrEP uptake [21].

The provision of oral PrEP in a research setting does offer some benefits. Participants may receive one-on-one education and counselling on oral PrEP including addressing any concerns, myths and fears. Regular study follow-up during the trial may facilitate the early recognition and management of oral PrEP related side effects. Study reimbursement and the provision of oral PrEP at no cost to participants removes financial and transport related barriers to PrEP access. Furthermore, research sites are less likely to have long waiting queues and may be better resourced than public sector health facilities. Therefore, while clinical trials may serve as a starting point for oral PrEP initiation and early use, sustainability and continued use can only be successful if participants who desire to continue using oral PrEP post study are able to access oral PrEP at local healthcare facilities.

Several models for oral PrEP delivery have been studied and ongoing studies are assessing novel and innovative methods for delivering PrEP, particularly models that simplify PrEP delivery. The integration of oral PrEP into routine family planning services was shown to be feasible in Kenya [23] and we found that in our study, many women continued to access family planning at clinics post study termination. HIV testing services may serve as an entry point for oral PrEP demand creation, however, despite some women in our study reporting accessing HIV testing services after exiting the study, oral PrEP was rarely discussed during HIV counselling. Antenatal and postnatal services may also serve as entry points for oral PrEP delivery and a study in Cape Town, South Africa found high rates of oral PrEP initiation and early continuation among this population [24]. In addition, differentiated models that include mobile and community-based platforms for oral PrEP delivery should be considered [14] as these might address facility-based barriers to oral PrEP access. A study in Cape Town found that delivering oral PrEP to AGYW using a community-based mobile health clinic was feasible and acceptable [25]. An additional community-based HIV testing and same-day PrEP initiation study is currently underway in South Africa [26].

Our study has some limitations. We interviewed a small number of women from one study site and therefore the generalizability of our study findings may be limited. It would have been useful to conduct a larger study and interview women from other trial sites, however due to costs and logistics, this study was limited to one trial site. Women were interviewed in English and not in their native language (isiZulu) and were interviewed by a study clinician, both of which could potentially bias our findings. While there is potential for social desirability and power relations to influence our findings, due to women being interviewed by a study clinician, this relationship could also be a strength and result in improved rapport. PrEP access and availability in South Africa have increased substantially over the last five years, and this needs to be considered when interpreting our study findings. Our study findings could be utilized by current and future clinical trials, especially as new effective HIV prevention products, such as longer acting PrEP products, become available. Furthermore, our findings may be useful for counselling participants in clinical trials about post-trial access and continued use of HIV prevention methods that might not yet be widely available in communities and facilities.

## Conclusions

Women who had initiated oral PrEP as part of HIV prevention standard of care during the ECHO Trial reported several barriers to oral PrEP access and moderate amounts of continued use following study exit. These barriers which included distance, transport costs, clinic operating hours, long queues, and limited oral PrEP availability frequently resulted in oral PrEP discontinuation. HIV testing services were frequently a missed opportunity to counsel and promote oral PrEP use. However, most women who had discontinued PrEP use reported wanting to use oral PrEP again, particularly if barriers to access could be alleviated or removed. The increased availability of oral PrEP in the public sector in South Africa could improve access to oral PrEP for trial participants following study termination.

## Data Availability

Access to data from the ECHO Study may be requested through submission of a research concept to icrc@uw.edu. The concept must include the research question, data requested, analytic methods, and steps taken to ensure ethical use of the data. Access will be granted if the concept is evaluated to have scientific merit and if sufficient data protections are in place. As of the time of publication, data access applications are in process with the governing institutional review boards of the ECHO Study to make de-identified data publicly available. Access to data from this study available upon request to i_beesham@yahoo.com.

## Abbreviations

AGYW: Adolescent girls and young women
ART: Antiretroviral therapy
ECHO Trial: Evidence for Contraceptive Options and HIV Outcomes Trial
HREC: Human Research Ethics Committee
MSM: Men who have sex with men
PrEP: Pre-exposure prophylaxis
SAMRC: South African Medical Research Council
STI: Sexually transmitted infection
WHO: World Health Organization

## References

[1] Gray GE, Bekker LG, Laher F (2021). Vaccine Efficacy of ALVAC-HIV and Bivalent Subtype C gp120-MF59 in Adults. N Engl J Med.

[2] ECHO Trial Consortium (2019). HIV incidence among women using intramuscular depot medroxyprogesterone acetate, a copper intrauterine device, or a levonorgestrel implant for contraception: a randomised, multicentre, open-label trial. Lancet.

[3] Corey L, Gilbert PB, Juraska M (2021). Two Randomized Trials of Neutralizing Antibodies to Prevent HIV-1 Acquisition. N Engl J Med.

[4] Brown B, Sugarman J. HPTN Ethics Guidance for Research: Revised February 2020. 2020.

[5] Joint United Nations Programme on HIV/AIDS and the World Health Organization. Ethical considerations in HIV prevention trials. Geneva. 2021. Licence: CC BY-NC-SA 3.0 IGO.

[6] South African Medical Research Council. Executive Summary of the Summit on the Standard of Care in Clinical Trials in Low-Middle Income Settings. 2017. Available at: https://www.samrc.ac.za/publications/executive-summary-summit-standard-care-clinical-trials-low-middle-income-settings. Accessed 07 Sep 2022. 10.1186/s13063-021-05754-z PMC8572437 34742340

[7] National Department of Health, South Africa. Guidelines for Expanding Combination Prevention and Treatment Options for Sex Workers: Oral Pre-Exposure Prophylaxis (PrEP) and Test and Treat (T&T). 2016. Available at: https://www.nicd.ac.za/assets/files/PrEP%20and%20TT%20Guidelines%20-%20Final%20Draft%20-%2011%20May%202016.pdf. Accessed 07 Sep 2022.

[8] Pillay D, Stankevitz K, Lanham M (2020). Factors influencing uptake, continuation, and discontinuation of oral PrEP among clients at sex worker and MSM facilities in South Africa. PLoS ONE.

[9] Department of Health, South Africa. Guidelines For The Provision Of Pre-Exposure Prophylaxis (Prep) To Persons At Substantial Risk Of HIV Infection. 2020. Available at: https://www.prepwatch.org/wp-content/uploads/2020/07/South-Africa-PrEP-Guidelines_Jan2020.pdf. Accessed 07 Sep 2022.

[10] Department of Health, South Africa. 2021 Updated Guidelines For The Provision Of Oral Pre-Exposure Prophylaxis (Prep) To Persons At Substantial Risk Of HIV Infection. 2021. Available at: https://www.knowledgehub.org.za/system/files/elibdownloads/2022-08/PrEP%20Guidelines%20Update%2012%20%20Nov%20%202021%20Final.pdf. Accessed 19 Sep 2022.

[11] AVAC PrEPWatch. Country overview: South Africa 2022. Available at: https://www.prepwatch.org/countries/south-africa/ Accessed 19 Sep 2022.

[12] Rousseau E, Katz AWK, O’Rourke S, et al. Adolescent girls and young women’s PrEP-user journey during an implementation science study in South Africa and Kenya. PLoS ONE. 2021;16(10):e0258542. 10.1371/journal.pone.0258542 PMC8516266 34648589

[13] Bhavaraju N, Shears K, Schwartz K (2021). Introducing the Dapivirine Vaginal Ring in Sub-Saharan Africa: What Can We Learn from Oral PrEP?. Curr HIV/AIDS Rep.

[14] Venter WDF (2018). Pre-exposure Prophylaxis: The Delivery Challenge. Front Public Health.

[15] Celum C, Hosek S, Tsholwana M (2021). PrEP uptake, persistence, adherence, and effect of retrospective drug level feedback on PrEP adherence among young women in southern Africa: Results from HPTN 082, a randomized controlled trial. PLoS Med.

[16] Beesham I, Welch JD, Heffron R (2020). Integrating oral PrEP delivery among African women in a large HIV endpoint-driven clinical trial. J Int AIDS Soc.

[17] Beesham I, Heffron R, Evans S (2021). Exploring the Use of Oral Pre-exposure Prophylaxis (PrEP) Among Women from Durban, South Africa as Part of the HIV Prevention Package in a Clinical Trial. AIDS Behav.

[18] Braun V, Clarke V (2006). Using thematic analysis in psychology. Qual Res Psychol.

[19] Department of Health, South Africa. Oral PrEP Update: March 20202. 2022. Available at: https://bhekisisa.org/wp-content/uploads/2022/04/PrEP-updated-18-March-2022.pdf. Accessed 19 Sep 2022.

[20] Gcwabe L. Matter of urgency: HIV prevention and support for women. 2022. Available at: https://health-e.org.za/2022/07/12/matter-of-urgency-hiv-prevention-and-support-for-women/. Accessed 19 Sep 2022.

[21] Muhumuza R, Ssemata AS, Kakande A (2021). Exploring Perceived Barriers and Facilitators of PrEP Uptake among Young People in Uganda, Zimbabwe, and South Africa. Arch Sex Behav.

[22] Gombe MM, Cakouros BE, Ncube G (2020). Key barriers and enablers associated with uptake and continuation of oral pre-exposure prophylaxis (PrEP) in the public sector in Zimbabwe: Qualitative perspectives of general population clients at high risk for HIV. PLoS ONE.

[23] Mugwanya KK, Pintye J, Kinuthia J (2019). Integrating preexposure prophylaxis delivery in routine family planning clinics: A feasibility programmatic evaluation in Kenya. PLoS Med.

[24] Joseph Davey DL, Mvududu R, Mashele N (2022). Early pre-exposure prophylaxis (PrEP) initiation and continuation among pregnant and postpartum women in antenatal care in Cape Town, South Africa. J Int AIDS Soc.

[25] Rousseau E, Bekker LG, Julies RF (2021). A community-based mobile clinic model delivering PrEP for HIV prevention to adolescent girls and young women in Cape Town, South Africa. BMC Health Serv Res.

[26] Medina-Marino A, Bezuidenhout D, Hosek S (2021). The Community PrEP Study: a randomized control trial leveraging community-based platforms to improve access and adherence to pre-exposure prophylaxis to prevent HIV among adolescent girls and young women in South Africa-study protocol. Trials.

